# Comparing the Quality of Direct-to-Consumer Telemedicine Dominated and Delivered by Public and Private Sector Platforms in China: Standardized Patient Study

**DOI:** 10.2196/55400

**Published:** 2024-11-14

**Authors:** Faying Song, Xue Gong, Yuting Yang, Rui Guo

**Affiliations:** 1 School of Public Health Capital Medical University Beijing China; 2 Beijing Luhe Hospital Capital Medical University Beijing China; 3 Hospital Management Research Institute Peking University Third Hospital Beijing China

**Keywords:** telemedicine, direct-to-consumer telemedicine, standardized patient, China, public, private, platform, objective evaluation, quality of care, effectiveness, safety, timeliness, regression model, management

## Abstract

**Background:**

Telemedicine is expanding rapidly, with public direct-to-consumer (DTC) telemedicine representing 70% of the market. A key priority is establishing clear quality distinctions between the public and private sectors. No studies have directly compared the quality of DTC telemedicine in the public and private sectors using objective evaluation methods.

**Objective:**

Using a standardized patient (SP) approach, this study aimed to compare the quality of DTC telemedicine provided by China’s public and private sectors.

**Methods:**

We recruited 10 SPs presenting fixed cases (urticaria and childhood diarrhea), with 594 interactions between them and physicians. The SPs evaluated various aspects of the quality of care, effectiveness, safety, patient-centeredness (PCC), efficiency, and timeliness using the Institute of Medicine (IOM) quality framework. Ordinary least-squares (OLS) regression models with fixed effects were used for continuous variables, while logistic regression models with fixed effects were used for categorical variables.

**Results:**

Significant quality differences were observed between public and private DTC telemedicine. Physicians from private platforms were significantly more likely to adhere to clinical checklists (adjusted β 15.22, *P*<.001); provide an accurate diagnosis (adjusted odds ratio [OR] 3.85, *P*<.001), an appropriate prescription (adjusted OR 3.87, *P*<.001), and lifestyle modification advice (adjusted OR 6.82, *P*<.001); ensure more PCC (adjusted β 3.34, *P*<.001); and spend more time with SPs (adjusted β 839.70, *P*<.001), with more responses (adjusted β 1.33, *P*=.001) and more words (adjusted β 50.93, *P*=.009). However, SPs on private platforms waited longer for the first response (adjusted β 505.87, *P*=.001) and each response (adjusted β 168.33, *P*=.04) and paid more for the average visit (adjusted β 40.03, *P*<.001).

**Conclusions:**

There is significant quality inequality in different DTC telemedicine platforms. Private physicians might provide a higher quality of service regarding effectiveness and safety, PCC, and response times and words. However, private platforms have longer wait times for their first response, as well as higher costs. Refining online reviews, establishing standardized norms and pricing, enhancing the performance evaluation mechanism for public DTC telemedicine, and imposing stricter limitations on the first response time for private physicians should be considered practical approaches to optimizing the management of DTC telemedicine.

## Introduction

Telehealth (telecare or telemedicine) has emerged as a sustainable alternative to traditional in-person visits, including clinician-to-clinician, clinician-to-patient, and patient-to-mobile health technology [1,2]. A virtual visit delivered by internet hospitals in China is equivalent to direct-to-consumer (DTC) telemedicine/telehealth [3], which directly provides medical services (appointments, consultations, e-prescriptions, drug delivery, health care management, etc) to patients by using information technology to extend medical resources from hospitals to the internet [4,5]. The COVID-19 pandemic has boosted telemedicine in medical care visits, and increasingly more people are beginning to use telemedicine [6,7]. According to the statistics of the China Internet Network Information Center, as of June 2023, the number of internet medical users in China reached 364 million, marking an increase of 1.62 million compared to December 2022 [8]. The demand for and the supply of DTC telemedicine in China have attained a novel level.

There are primarily public brick-and-mortar hospital-sponsored and private enterprise–sponsored internet hospitals in China’s DTC telemedicine market [3]. Public DTC telemedicine is considered a nonprofit medical service, and its workforce comprises practicing physicians in public hospitals [9]. Its charges must be implemented in accordance with the government’s price policy, and the government pays the input costs. Public DTC telemedicine assigns patients to doctors and mainly provides basic medical services. In 2014, the Second Provincial Hospital of Guangdong established the first internet hospital in China [10]. In 2016, the first public tertiary hospital campus–sponsored internet hospital (the First Affiliated Hospital of Zhejiang University) was launched [11]. In 2018, the State Council of China issued the “Internet Plus Healthcare of 2018” guideline [12], and the National Health Commission accordingly issued specific regulation schemes for online medical diagnosis and treatment in 2018 [13], which have brought about the rapid development of DTC telemedicine [6], especially public DTC telemedicine. According to statistics, approximately 70% of internet hospitals in China have been initiated by public hospitals (public DTC telemedicine) [14].

Private DTC telemedicine operates as a self-contained for-profit institution, with services provided by platform physicians or doctors with multiple practices. The pricing can be carried out according to the market law [9]. Around 2010, private enterprises, such as WeDoctor and Haodf, took the lead in launching online health care, providing physician comments/ratings, appointments and referrals, and postconsultation management, which is the prototype of China’s DTC telemedicine. These enterprises use physicians’ brands to attract patients, which can better address patient demands and offer a broader spectrum of services. The integration of advanced technologies, such as artificial intelligence and big data analytics, has further enhanced the capabilities of these platforms, enabling more personalized and precise health care services.

Theoretically, opponents of the private sector argue that profit-driven private health care providers can manipulate services to maximize profits rather than quality [15,16]. In contrast, a competing theory claims that private health care providers can use more up-to-date and flexible management to improve quality [17]. With the rapid development of DTC telemedicine in China, it is a priority to clarify the difference in health care quality between public and private health care providers, which can provide some information and inspiration for patients when choosing services. However, the existing quality evaluations of private and public medical services mainly focus on face-to-face services, and the evidence is insufficient and conflicting [18-21]. There is no nationally representative sample, as previous studies have compared the quality of private and public primary health care within specific Chinese cities. Internationally, 2 earlier reviews [22,23] of 80 and 102 studies, respectively, came to opposite conclusions regarding the relative quality of the 2 sectors. One concluded that the private sector is more responsive, puts in more effort, and can prioritize clients’ needs [22], while the other failed to support the claim that the private sector is generally more efficient, responsible, or medically effective than the public sector [23]. Another 2 reviews (including the latest 2017 one) [24,25] added no certainty to the debate. Regarding DTC telemedicine, some scholars have evaluated its subjective quality based on patient perception and recall-based surveys rather than actual clinical ability; other scholars have explored its objective quality based on patient data and the standardized patient (SP) approach [26,27]. For instance, Resneck et al [28] contended that although telemedicine may enhance the availability of high-value health care, its accuracy of diagnoses and quality of prescriptions and treatment recommendations have not met expectations because of fragmentation. Gong et al [29] assessed the quality of DTC telemedicine services using an SP approach. They discovered that doctors with better response times and more words are significantly more likely to adhere to clinical checklists and provide accurate diagnoses, appropriate prescriptions, and lifestyle modification advice. Crucially, there is yet no evidence from large samples of quality differences between public and private telemedicine.

Studies have suggested that comparing services provided by the public and private sectors needs to address 3 significant barriers: a clear definition of quality, an accurate measurement of quality, and a sufficient adjustment for the case mix [30]. For this study, we referred to the framework developed by the Institute of Medicine (IOM) [31] and the specific measurement indicators proposed by Xu et al [30] based on this model to define quality. Furthermore, we suggested using SPs to measure quality and control the case mix accurately. This method has gained rapid acceptance for quality measurement in countries worldwide [32-34]. The SP approach enables researchers to compare the care that is provided against predetermined national standards of care; avoids Hawthorne effects, whereby doctors change their behavior because they know they are being observed; and allows researchers to control for potential confounding arising from differences in the case mix and patient recall bias [35].

Text-based consultation is among the most frequently used DTC telemedicine services in China. Therefore, we used SPs to collect and compare the quality between public and private internet text-based consultations. This study is the first large sample of evidence to evaluate and compare the quality of public and private DTC telemedicine services using an SP approach and the IOM framework in the context of China. In anticipation, our findings will inform policy makers in China and similar countries that provide public/private DTC telemedicine.

## Methods

### Case Selection

Each SP presented 1 of 2 medical cases, urticaria or childhood diarrhea of an absent child. Some scholars have proposed 10 questions to consider when assessing suitability as an SP case [36]. The specific reasons for choosing the 2 cases are presented in Multimedia Appendix 1. In general, these diseases (1) have a high incidence in China, (2) are easy to be portrayed online through pictures and texts, (3) can be diagnosed preliminarily without an offline invasive examination, and (4) have national guidelines and clear clinical treatment pathways (medication, patient education, etc).

### Study Design and Setting

**Figure 1:**
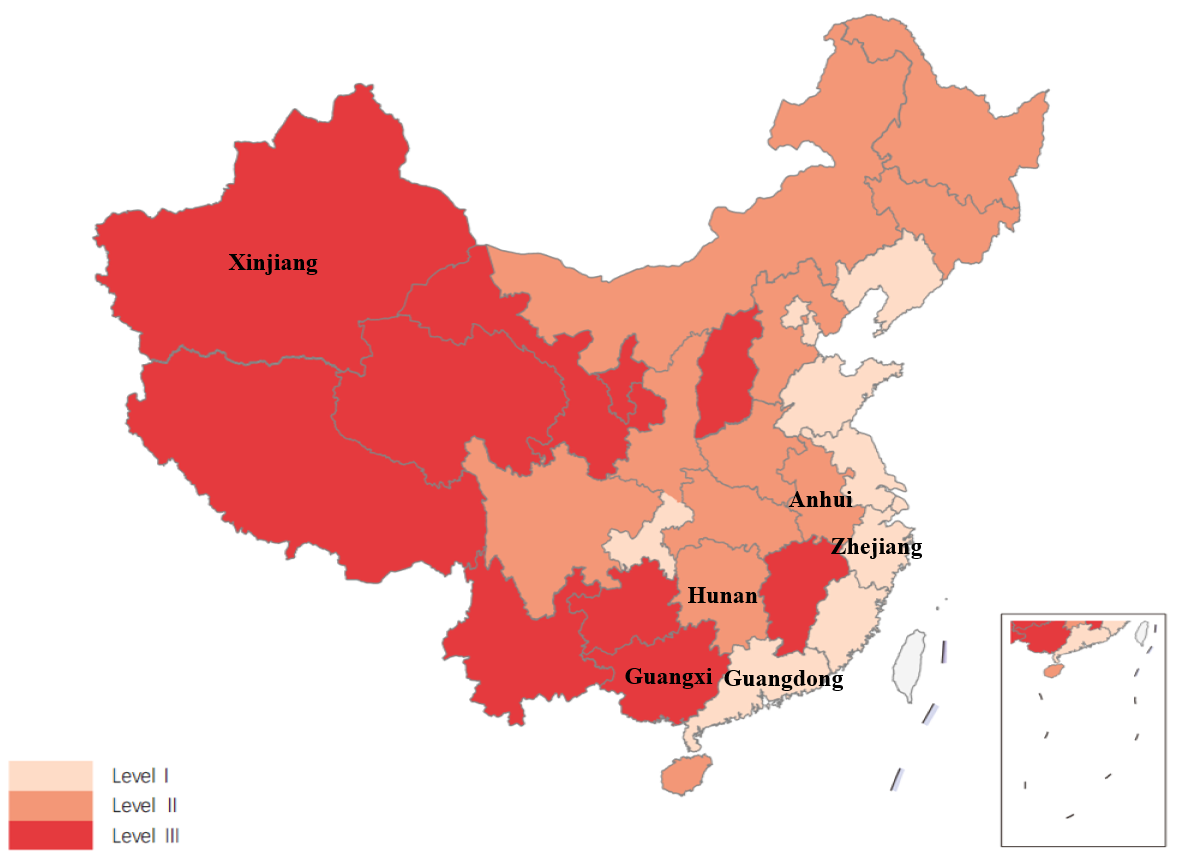
Six selected sample provinces on the map of China.

We considered creating a nationally representative probability sample of China’s DTC telemedicine providers in tertiary hospitals. The provinces have been divided into 3 levels based on a comprehensive ranking that considers both economic development (judging from the real gross domestic product [GDP] per capita) and health status (judging from life expectancy at the provincial level), with each factor weighted equally (Figure 1), which also corresponds to the regional distribution (eastern, central. and western) [37]. Ultimately, for feasibility reasons, we chose 6 provinces (Guangdong, Zhejiang, Hunan, Anhui, Guangxi, and Xinjiang) to represent China. Multimedia Appendix 2 presents the process of dividing the provinces into 3 levels.

In each provincial capital city, we selected medical institutions that met all our following criteria:

- Public sector platforms: (1) tertiary public hospitals with (2) independent platforms based on offline physical hospitals established to provide DTC telemedicine, (3) dermatology/pediatrics DTC telemedicine services, and (4) asynchronous graphic consultations
- Private sector platforms: (1) DTC telemedicine services on Haodf (one of China’s earliest and most popular private online medical platforms) while practicing at physical tertiary public hospitals, (2) dermatology/pediatrics DTC telemedicine services, and (3) asynchronous graphic consultations

Dermatologists and pediatricians from eligible institutions constituted the sampling frame.

The sample size was calculated according to the following formula:



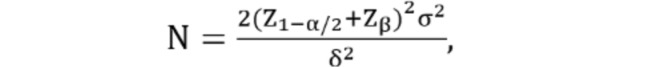



where σ is the SD from the population, δ is the difference between the 2 groups that is clinically significant, and N is the sample size for each setting. Assuming a 5% type I error (α) and 95% power (1 – β), according to previous research [38], δ=0.15 and σ=0.31, the sample size for 1 setting should reach 110, that is, the visits should be more than 110 times for each setting. Given that some doctors may fail to visit (unable to conduct a second visit, the SP is denied or identified on the spot, etc) or the quality of the records may be low, and other reasons, the sample size was expanded by 35%, so the sample size of this study was 150 times. Therefore, 600 visits for 600 doctors were implemented in this study.

**Figure 2:**
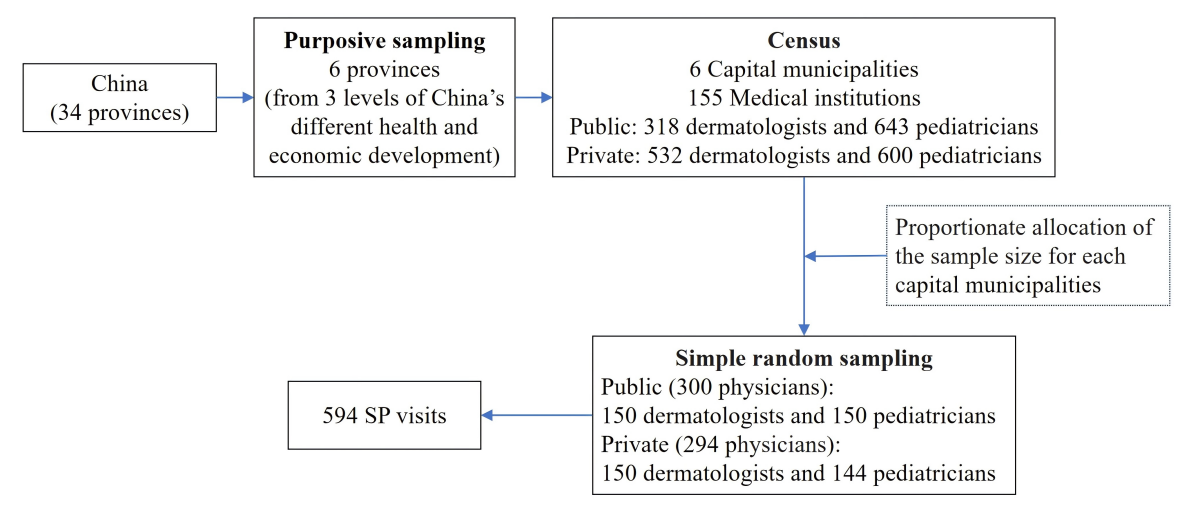
Sampling procedure. SP: standardized patient.

Proportionate sampling was used to determine the weight of each provincial capital city stratum. The weight of the stratum was the proportion of the population contained in that stratum, and the population comprised the permanent residents of the Seventh National Census in each city. The definition of telemedicine areas was based on the location of the hospital where the doctors practice offline. We calculated that 45, 28, 24, 22, 21, and 10 physicians should be included in the provincial capital city in each setting. However, due to the limitation of the total number of pediatricians in the capital city of Guangxi Province on Haodf, only 15 physicians were included in the setting of pediatrics on private sector platforms. A total of 594 physicians were eventually included as respondents in this study. Figure 2 summarizes the sampling procedure.

### Standardized Patient Procedure

#### Script and Checklist

We developed a detailed script that consulted doctors and professors with extensive experience for the SPs to use in their visits with the doctors. In both cases, urticaria and childhood diarrhea, preliminary scripts based on established disease guidelines were distributed to 3 pediatricians and 3 dermatologists to improve professionalism and accuracy. Two professors were assigned to each script for fluency and colloquial language. The final scripts covered all possible questions a physician may ask and the SPs’ answers during the consultation. Each script included (1) a detailed background story, (2) an opening statement, (3) an illness history presented in question-and-answer format, (4) the language to insist on diagnosis/prescription/lifestyle advice if not given, and (5) a peroration. The checklists of specific cases provide recommended questions that a physician should ask or perform during the interactions (the condensed scripts and checklists are provided in Multimedia Appendix 3).

#### Recruitment and Training of Standardized Patients

Recruitment and training protocols ensured that the SPs conformed closely to the doctors’ regular patient populations. All SPs were recruited from a college following these essential criteria: (1) SPs must be in good physical condition without confounding symptoms, and (2) students of medicine were not recruited as SPs because their medical knowledge and behaviors might affect the diagnosis and treatment of the counterpart physician. In total, 10 SPs were recruited.

A cross-disciplinary team of professors, medical experts, and investigators trained the SPs for 1 week to consistently perform in each encounter according to case scenarios and scripts. This extensive training led to low detection rates of SPs by the sampled doctors. No doctor voiced any suspicions, so the detection rate was, thus, considered 0.

#### Standardized Patient Visit

The 10 SPs were randomly allocated and visited each physician independently during clinic or nonclinic hours from July 4 to August 5, 2022 (n=5, 50%, SPs describing urticaria and n=5, 50%, SPs describing childhood diarrhea). Each encounter was performed according to the scripts developed. The SPs saved mobile phone screenshots to record the interactions with physicians, allowing us to accurately evaluate interactions without recall bias.

#### Standardized Patient Exit Survey

SPs completed an exit survey with a structured questionnaire immediately after the visit based on the screenshots to extract information about what each doctor had done, which included (1) a technical quality checklist (adherence to the checklist, defined as the proportion of recommended questions that were asked during the SP consultation, the accuracy of diagnosis, the appropriateness of the prescription, and the lifestyle modification advice), (2) a doctor-patient communication scale (patient-centeredness [PCC]), and (3) a list that recorded the total cost and timeliness of services (time waiting for the first response, time waiting for each response, time for consultation, total number of the doctor’s responses, and total words in all of the doctor’s responses). The case developers checked the screen recordings to ensure the quality of the SPs’ performance and the accuracy of filling out the exit questionnaire at each visit.

### Evaluating the Quality of DTC Telemedicine

We measured the quality of DTC telemedicine based on various aspects of the IOM framework (Tables 1 and 2): (1) effectiveness and safety (adherence to the checklist, accuracy of diagnosis, appropriateness of the prescription, and lifestyle modification advice), (2) PCC (following the Patient Perception of Patient-Centeredness [PPPC] rating scale) [39,40], (3) timeliness (time waiting for the first response, time waiting for each response, time for consultation, total number of the doctor’s responses, and total words in all of the doctor’s responses), and (4) efficiency.

**Table 1:** Outcome variables used in this study and their definitions.

Variables		Definition	Type	Coding	Source
Effectiveness and safety					
	Adherence to the checklist	Adherence to the case-specific checklist in China’s national clinical guidelines [41,42]	Continuous	0-1	SP^a^ checklist
	Accurate diagnosis	Urticaria, not allergy, erythema annulare, popular urticaria, or other diseases (viral diarrhea, infective diarrhea)	Dichotomous	0: no; 1: yes	SP checklist
	Appropriate prescription	A definite second-line medication prescribed, with clearly defined course duration and dosage	Dichotomous	0: no; 1: yes	SP checklist
	Lifestyle modification advice	Lifestyle modification advice provided	Dichotomous	0: no; 1: yes	SP checklist
PCC^b^					
	PCC1: exploring illness experiences	Table S4 in Multimedia Appendix 4	Continuous	1-5	PPPC^c^ rating scale
	PCC2: understanding the whole person	Table S4 in Multimedia Appendix 4	Continuous	0-3	PPPC rating scale
	PCC3: finding the common ground	Table S4 in Multimedia Appendix 4	Continuous	3-17	PPPC rating scale
Timeliness					
	Time waiting for the first response	Time interval between the patient’s first question and the doctor’s first response	Continuous	Minutes	Time record
	Time waiting for each response	Average time interval between each patient’s question and the doctor’s response	Continuous	Minutes	Time record
	Time for consultation	Time interval between the patient’s first question and the doctor’s last response	Continuous	Minutes	Time record
	Total number of the doctor’s responses	Total number of times of doctor responds in 1 encounter	Continuous	Times	Records
	Total words in all of the doctor’s responses	Total number of words spoken by the doctor in 1 encounter	Continuous	Words	Records
Efficiency					
	Total cost	Charge of each visit	Continuous	RMB^d^	Cost record

^a^SP: standardized patient.

^b^PCC: patient-centeredness.

^c^PPPC: Patient Perception of Patient-Centeredness.

^d^RMB: renminbi.

**Table 2:** Other variables used in this study and their definitions.

Variables			Definition		Type		Coding		Source
Exposure									
	Ownership type	Private and public		Dichotomous		1: public; 2: private		Records	
Possible clinician factors influencing quality									
	Region	Doctor’s practice place		Ordinal		1: level Ⅰ; 2: level II; 3: level III		Records	
	Gender	Doctor’s gender		Dichotomous		0: male; 1: female		Records	
	Institutional type	Type of institution where the doctor works		Dichotomous		0: traditional Chinese medicine hospital; 1: general hospital		Records	
	Title	Doctor’s title		Ordinal		1: associate chief physician; 2: chief physician; 3: resident physician; 4: attending physician		Records	
Possible patient factors influencing quality									
	Timing of the visit	Whether SPs^a^ visit the doctor during clinic hours		Dichotomous		1: clinic hours; 2: nonclinic hours		Records	

^a^SP: standardized patient.

### Statistical Analysis

Our analysis unit was the interaction between physicians and SPs. Descriptive statistics identified the sociodemographic characteristics of the final sample. We used the median and interquartile distances to describe continuous variables and used the number and percentage to describe categorical variables. The Wilcoxon rank-sum test was performed to assess the differences in service quality. Ordinary least-squares (OLS) regression models with fixed effects were used for the continuous variables and logistic regression models with fixed effects for the categorical variables, reporting coefficients (β) with the accompanying clustering robust standard error in OLS regressions and the odds ratio (OR) with the accompanying clustering robust standard error in logistic regressions. All regressions were adjusted for critical covariates (region, sex, institution, and title). We performed statistical analyses using Stata version 15 and considered *P*<.05 statistically significant (2-tailed).

### Sensitivity Analysis

We recompared the quality of public and private DTC telemedicine services in 2 cases: the first included all interactions contributed by SPs acting as patients with urticaria, and the second included all interactions contributed by SPs acting as patients with childhood diarrhea. The quality of services in public and private DTC telemedicine was assessed for all cases.

### Ethical Considerations

In this study, there was no personal information of a single patient because we used SPs to present both urticaria and childhood diarrhea. In addition, our study did not involve the personal information of a single doctor but focused on the overall distribution of the sociodemographic characteristics of a sample of doctors. Informed consent was obtained from all SPs. However, to avoid the Hawthorne effect, the informed consent of physicians was not required for this study. Ethical approval for this study was obtained from the Ethics Committee of Capital Medical University (2021SY074).

## Results

### Basic Characteristics

Consultations were categorized as effective or ineffective depending on whether the doctor provided a diagnosis, a prescription, or a referral recommendation. In total, 321 effective consultations were included. Of them, interactions of public and private sectors accounted for 54.5% (175/321) and 45.5% (146/321), respectively. In addition, 173 (53.9%) cases of urticaria and 148 (46.1%) cases of childhood diarrhea were analyzed. Furthermore 220 (68.5%) physicians were from general hospitals, most physicians were female (n=207, 64.5%), were located in a level Ⅰ city (n=161, 50.2%), and had the title of attending physician (n=124, 38.6%) or associate chief physician (n=109, 34%). In 169 (52.6%) interactions, SPs visited the physician during clinic hours, and in the other 152 (47.4%) interactions, they visited the physician during nonclinic hours. Interactions varied by case, type of institution, and physician gender between public and private sectors; however, there were no differences in other variables. More details can be found in Table 3.

**Table 3:** Characteristics of interactions between physicians and SPs^a^.

Characteristics		Total (N=321), n (%)	Public sector (n=175), n (%)	Private sector (n=146), n (%)
Case (*χ*^2^_1_=4.387, *P*=.04)				
	Urticaria	173 (53.9)	85 (48.6)	88 (60.3)
	Childhood diarrhea	148 (46.1)	90 (51.4)	58 (39.7)
Region (*χ*^2^_2_=1.535, *P*=.46)				
	Level Ⅰ	161 (50.2)	93 (53.1)	68 (46.6)
	Level Ⅱ	103 (32.1)	54 (30.9)	49 (33.6)
	Level Ⅲ	57 (17.8)	28 (16.0)	29 (19.9)
Type of institution (*χ*^2^_2_=13.192, *P*=.001)				
	General hospital	220 (68.5)	105 (60.0)	115 (78.8)
	Specialized hospital	36 (11.2)	26 (14.9)	10 (6.8)
	Traditional Chinese medicine hospital	65 (20.2)	44 (25.1)	21 (14.4)
Physician gender (*χ*^2^_2_=7.393, *P*=.03)				
	Female	207 (64.5)	124 (70.9)	83 (56.8)
	Male	95 (29.6)	44 (25.1)	51 (34.9)
	Missing	19 (5.9)	7 (4.0)	12 (8.2)
Physician title (*χ*^2^_3_=3.493, *P*=.32)				
	Chief physician	47 (14.6)	23 (13.1)	24 (16.4)
	Associate chief physician	109 (34.0)	61 (34.9)	48 (32.9)
	Attending physician	124 (38.6)	73 (41.7)	51 (34.9)
	Resident physician	41 (12.8)	18 (10.3)	23 (15.8)
Timing of the visit (*χ*^2^_1_=0.414, *P*=.52)				
	Clinic hours	169 (52.6)	95 (54.3)	74 (50.7)
	Nonclinic hours	152 (47.4)	80 (45.7)	72 (49.3)

^a^SP: standardized patient.

### Quality of DTC Telemedicine in Private and Public Sectors

**Figure 3:**
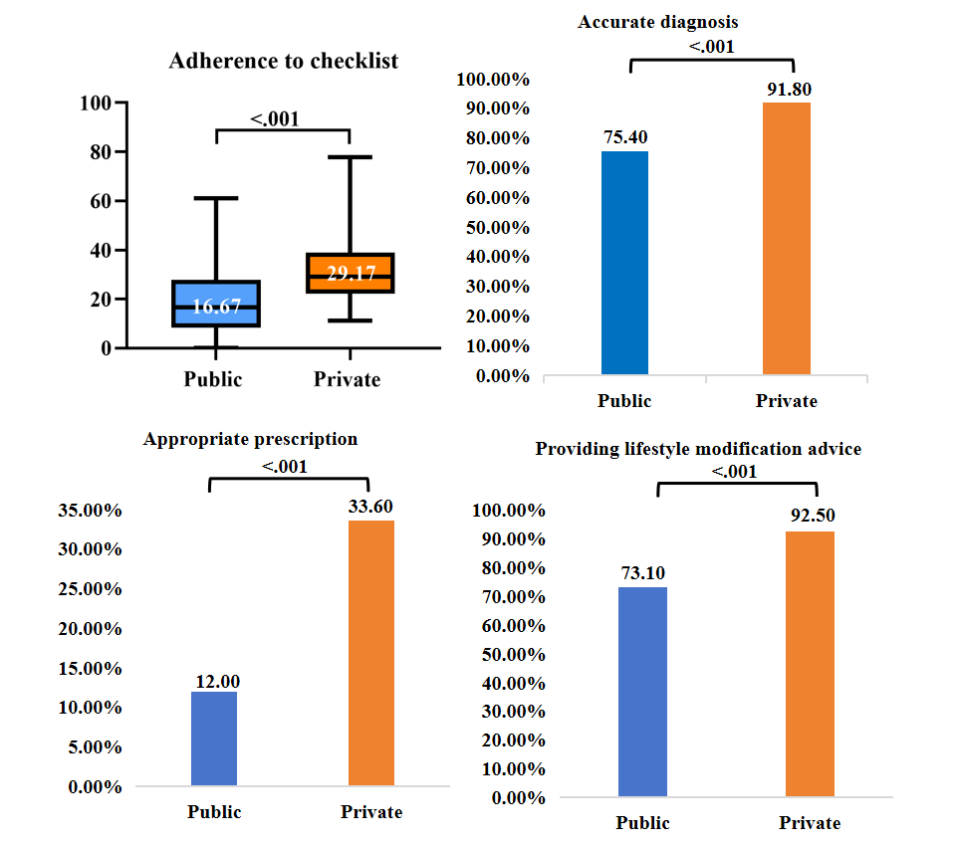
Effectiveness and safety of DTC telemedicine provided by public and private sectors. DTC: direct to consumer.

**Figure 4:**
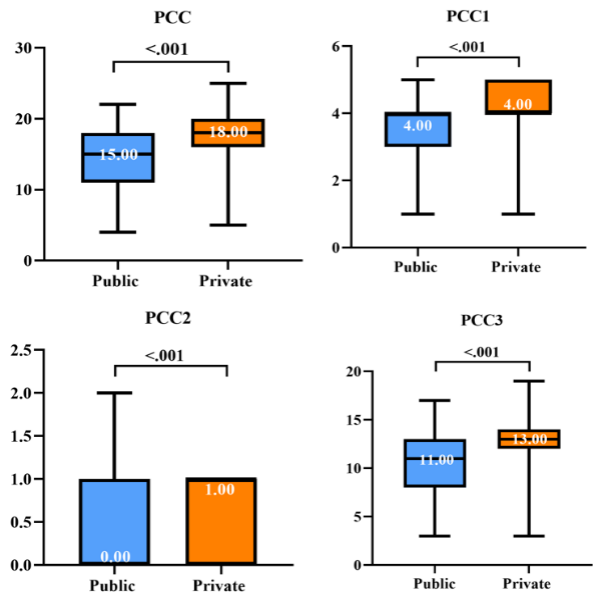
PCC of DTC telemedicine provided by public and private sectors. DTC: direct to consumer; PCC: patient-centeredness.

**Figure 5:**
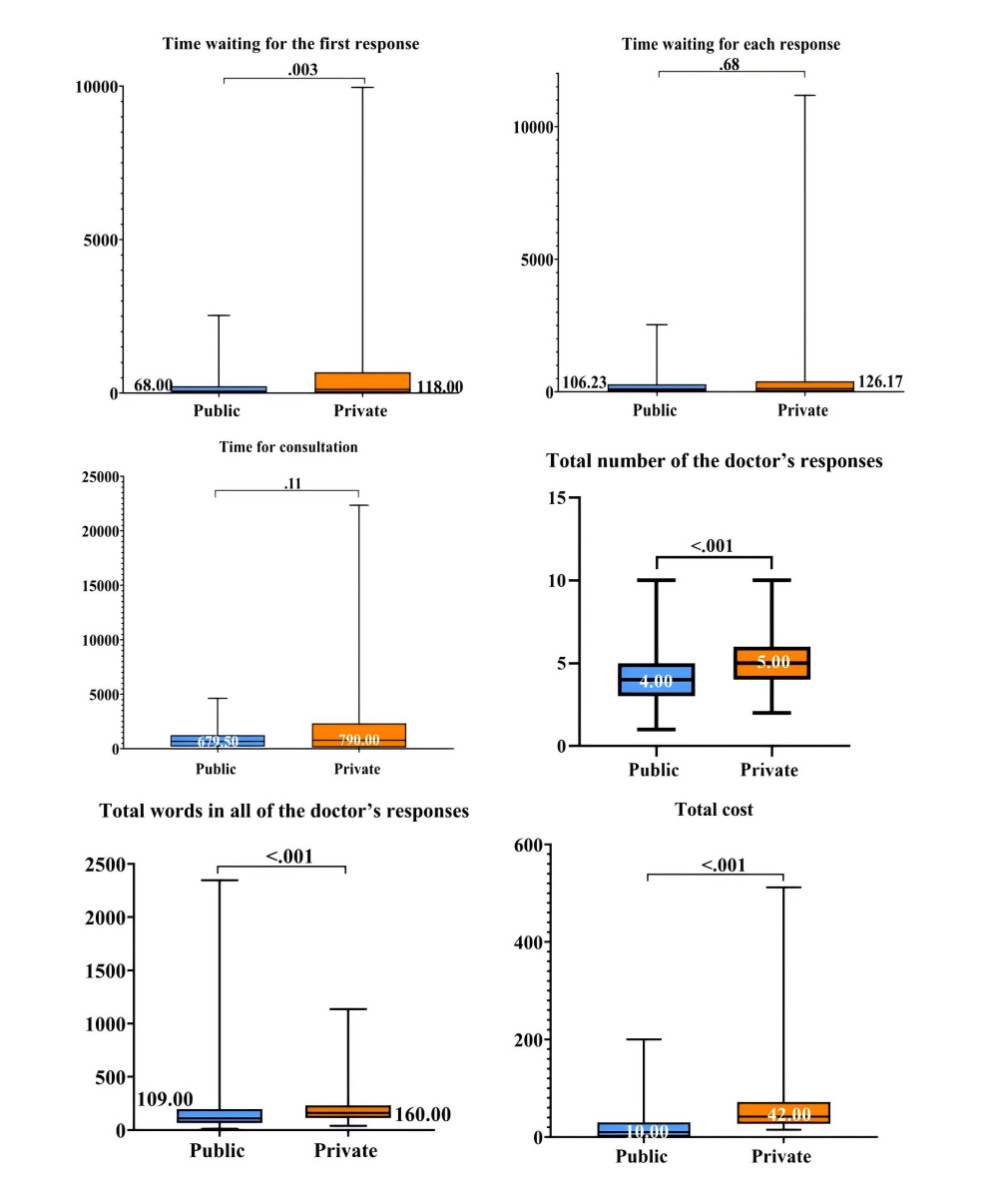
Timeliness and efficiency of DTC telemedicine provided by public and private sectors. DTC: direct to consumer.

Figures 3-5 and Table S5 in Multimedia Appendix 5 describe the quality of DTC telemedicine provided by the public and private sectors. The median percentage (29.17%) of items completed for checklists in the private sector was higher than that (16.67%) in the public sector (*P*<.001). Furthermore, the effectiveness and safety of consultations provided by private sector platforms were consistently higher than those provided by public hospitals (accurate diagnosis: 91.8% vs 75.4%; appropriate prescription: 33.6% vs 12.0%; lifestyle modification advice: 92.5% vs 73.1%). The median PCC scores were 15 (IQR 11-18) and 18 (IQR 16-20) for the public and the private sector, respectively. The median total number of doctor’s responses from the private sector was 5 (IQR 4-6), which was more in than that in the public sector (median 4, IQR 3-5; *P*<.001). The median total number of words in the doctor’s responses in the private sector was 160 (IQR 114.00-232.25), which was also more than that in the public sector (median 109, IQR 66-198; *P*<.001). In contrast, public sector physicians had shorter waiting times for the first response than private sector physicians (median 68, IQR 9-222, vs median 118, IQR 22.25-672.75). The median cost of each interaction was lower in the public sector (median 10, IQR 0-30) than in the private sector (median 42, IQR 27-72; *P*<.001).

### Association Between the Models and the Quality of DTC Telemedicine

Table 4 shows the association between the models and the quality of DTC telemedicine. After adjustments were made for case, region, type of institution, physician gender, physician title, and timing of the visit, significant quality differences were observed between the public and private sectors. The results showed that physicians from private platforms were significantly more likely to adhere to clinical checklists (adjusted β 15.22, *P*<.001); provide an accurate diagnosis (adjusted OR 3.85, *P*<.001), an appropriate prescription (adjusted OR 3.87, *P*<.001), and lifestyle modification advice (adjusted OR 6.82, *P*<.001); ensure more PCC (adjusted β 3.34, *P*<.001); and spend more time with SPs (adjusted β 839.70, *P*<.001), with more responses (adjusted β 1.33, *P*=.001) and more words (adjusted β 50.93, *P*=.009). However, SPs on private platforms waited longer for the first response (adjusted β 505.87, *P*=.001) and each response (adjusted β 168.33, *P*=.04) and paid more for the average visit (adjusted β 40.03, *P*<.001).

**Table 4:** Effects of models on the quality of DTC^a^ telemedicine^b^.

Characteristics		Public sector adjusted β/OR^c^ (SE)	Private sector adjusted β/OR (SE)	*P* value
Effectiveness and safety				
	Adherence to the checklist	Reference	15.22 (1.63)	＜.001
	Accurate diagnosis	Reference	3.85 (1.46)	＜.001
	Appropriate prescription	Reference	3.87 (1.41)	＜.001
	Lifestyle modification advice	Reference	6.82 (2.57)	＜.001
PCC^d^				
	PCC	Reference	3.34 (0.34)	＜.001
	PCC1	Reference	0.60 (0.08)	＜.001
	PCC2	Reference	0.33 (0.14)	.04
	PCC3	Reference	2.41 (0.34)	＜.001
Timeliness				
	Time waiting for the first response	Reference	505.87 (95.85)	.001
	Time waiting for each response	Reference	168.33 (68.89)	.04
	Time for consultation	Reference	839.70 (151.31)	＜.001
	Total number of the doctor’s responses	Reference	1.33 (0.26)	.001
	Total words in all of the doctor’s responses	Reference	50.93 (15.30)	.009
Efficiency				
	Total cost	Reference	40.03 (4.12)	＜.001

^a^DTC: direct to consumer.

^b^The variables were controlled as follows: case, region, type of institution, physician gender, physician title, and timing of the visit, and SE was the clustering robust standard error.

^c^OR: odds ratio.

^d^PCC: patient-centeredness.

### Sensitivity Analysis Outcomes

Table S6 in Multimedia Appendix 6 shows the sensitivity analysis results for urticaria. The results of the regressions were close to our initial analysis results, except for the time waiting for each response (*P*=.10) and the total words in all of the doctor’s responses (*P*=.10), reporting no statistical effect.

Table S7 in Multimedia Appendix 6 shows the sensitivity analysis results for childhood diarrhea. The results of the regressions differed from the entire sample in prescriptions (*P*=.10), time waiting for the first response (*P*=.07), time waiting for each response (*P*=.44), and time for consultation (*P*=.07), presenting no statistical effect.

## Discussion

### Principal Findings

This study is the first large sample of evidence to evaluate and compare the quality of DTC telemedicine between public and private health care providers using an SP approach. Previous studies have suggested that quality studies have rarely been based on large random samples, sufficient numbers of tracer conditions, and gold-standard assessment tools [32]. Our study conducted a more thorough and valid comparison between private and public health care providers, with an adequately powered and representative sample of doctors. We found the private sector fares better regarding technical quality indicators, such as adherence to clinical checklists, PCC, and total numbers of and words in doctors’ responses. However, the public sector performed better in the time waiting for the first response and the total cost. The results could provide a strategic reference for improving the quality of public and private DTC telemedicine in China.

#### Advantages of Private DTC Telemedicine Quality

Compared to public DTC telemedicine, private doctors were more likely to adhere to clinical checklists, give a higher proportion of accurate diagnoses and appropriate prescriptions, and provide lifestyle modification advice. This aligns with a prior study comparing the quality of tuberculosis care in offline public and private health care facilities in Mumbai, India, where private doctors asked more medical history questions and were more likely to complete more clinical checklists and history collection programs [35]. However, it contradicts the findings of 2 studies comparing primary health care services sectors in China [43]. The differences in study conclusions could be attributed to discrepancies in study settings, diseases, and physician populations. Reliability and ease of use are critical for physicians in telemedicine [44]. DTC telemedicine in the public sector in China started later than in the private sector, increasing mainly during the COVID-19 pandemic. The incomplete construction and mechanism of DTC telemedicine platforms in the public sector may affect the doctors’ experience, ultimately hindering their efforts in consultations. Doctors who exert more effort perform more clinical checklist recommendations and diagnose more precisely and can also avoid unnecessary care [45]. However, more empirical studies are required to test specific reasons.

Furthermore, private DTC telemedicine providers exhibited greater PCC than those in the public sector, contradicting a study on community health centers [43]. A possible interpretation is that compensation mechanisms, education levels, and relevant rules and regulations are all factors that affect doctors’ behavior [46,47]. Reward and punishment systems, the professional environment, and reactions to the market competition also impact doctors’ behavior. DTC telemedicine in the private sector amplifies the role of a doctor’s brand in attracting patients. The private sector has established a sound and standardized postvisit evaluation mechanism, which displays a doctor’s comprehensive score and online reviews on the doctor’s homepage. Online reviews create an easily accessible digital word of mouth for patient-perceived care quality [48]. A recent national survey in the United States showed that 59% of participants referred to online reviews when choosing a physician [49]. Patients’ ratings of doctors depend on whether they have received tangible help from those doctors and have had an excellent medical experience. Therefore, doctors who explore more patients’ disease experiences, family and social backgrounds, and doctor-patient consensus can obtain high scores and build a good brand and reputation to attract more patients.

In addition, responses from private sector doctors were more detailed and frequent, meaning they provided more numbers of and words in responses. A large proportion of the difference may be due to financial incentives tied to service prices. Internet medical service items and prices are important factors affecting the patient acceptance and participation enthusiasm of doctors and medical institutions [50]. The lower cost of DTC telemedicine in public sectors leads to lower motivation. The cost of a single consultation on the Haodf platform belongs to the doctor in full [51]. It allows pricing according to the quality of service, which promotes the doctor’s active service provision behavior. Doctors provide better online services in response to patients’ financial efforts [52].

The public sector should learn some practices from the private sector. First, a standardized postvisit evaluation mechanism should be established to show patients’ disease experience and encourage doctors to be more patient centered. Second, corresponding norms and price standards for DTC telemedicine should be formulated, and the performance incentive evaluation mechanism should be improved so that doctors with different titles can fully realize their labor value. One hospital in Shenzhen pioneered a successful strategy of holding weekly meetings, while using DTC telemedicine fees as a performance reward to increase doctors' enthusiasm [53].

#### Advantages of Public DTC Telemedicine Quality

The time waiting for the first response differed significantly between the 2 sectors, with the public sector performing better. One possible explanation is that nearly 60% of public sector consultations require doctors to receive consultations within 24 hours and about 22% require consultations within 48 hours, while in the private sector, doctors can respond to graphic consultations initiated by patients after 1 week. This results in a shorter wait time for the public sector doctor’s first response (ie, a timelier response). Establishing regulations to govern the response time for initial DTC telemedicine consultations is crucial in ensuring a prompt reply. For instance, health care professionals must respond within 48 hours.

The private sector had a higher total cost than public DTC telemedicine. This may be due to the different pricing strategies of the 2 models. The public sector primarily operates as a nonprofit and adheres to government-guided fee schedules, with some institutions even providing free consultation services. In this study, free consultation accounted for 28.6% (50/175) of effective consultations in the public sector. The private sector mainly operates for profit, implementing market-regulated prices, where the cost of a single consultation can reach up to Chinese yuan (CNY) 512 (~US $72).

#### Disadvantages of Public and Private DTC Telemedicine Quality

We further analyzed several poor aspects of DTC telemedicine services. DTC telemedicine had poor rates of appropriate prescribing and adherence to clinical checklists, at 21.8% and 25%, respectively, with a low PCC2 score. Previous studies have found that although most physicians acknowledge the positive effects of guidelines, understanding and adhering to the guidelines still need to be improved. Factors related to guidelines, individuals, patients, organizations, and the environment conspire to contribute to noncompliance [54]. The results of this study reflect that most prescriptions do not have the necessary 3 elements (definite second-line medication, clearly defined course duration, and dosage). One interpretation is that DTC telemedicine may exacerbate uncertainty in doctors’ understanding of patients’ illnesses. To avoid risk, doctors may make vague prescribing recommendations [55]. Regarding PCC2, doctors are busy, pay more attention to diseases when facing patients, and make judgments on time. The separation of the biological and social nature of patients causes doctors to pay little attention to the social background of patients [56].

These findings highlight several strategies that may help improve the quality of DTC telemedicine. On the one hand, it is essential that organizations, especially public DTC telemedicine institutions, add advisory templates with clinical guidelines, such as significant symptoms and dosage, on the platforms. These can also help save time because doctors complete e-consults outside their commonly designated work [57]. Doctors argue that using the current system for documentation is complex and inefficient and creates a time burden. On the other hand, public and private sectors can reestablish scientific and reasonable doctor scheduling and collect information on the time distribution when patients initiate consultations. For instance, a hospital in western China discovered online prescription searches had 2 distinct peaks at 9:00 AM and 8:00 PM [58].

### Limitations

This study should be interpreted in the context of several potential limitations. Due to the study area, the conclusion may need more generalizability. First, we only carried out an SP survey on childhood diarrhea and urticaria. Both diseases do not require invasive examinations. Considering that the diagnosis and treatment of each disease are quite different, the study’s conclusion may not be extrapolated to other diseases. Second, even though we surveyed 6 cities in the context of DTC telemedicine, we still need to be cautious about generalizing the findings to the overall quality of DTC telemedicine. Moreover, our research was conducted only on 1 of the earliest and most popular private platforms in China. We will experiment with a broader sample of health care systems to address some of these challenges.

### Conclusion

This study is the first large sample of evidence to evaluate and compare the quality of DTC telemedicine between public and private sector doctors using the SP approach in China. The study added evidence suggesting that comparisons of the quality of DTC telemedicine services provided by the public and private sectors need to consider different quality indicators. Compared with public DTC telemedicine, private DTC telemedicine providers are more likely to adhere to clinical checklists; providing a higher proportion of accurate diagnoses, appropriate prescriptions, and lifestyle modification advice; and being more patient centered. However, DTC telemedicine in the public sector has lower costs and faster responses. Bearing in mind the findings, this study has highlighted several strategies that might be helpful to improve the quality of DTC telemedicine. First, DTC telemedicine in the public sector should make efforts to enhance the patient experience by developing online reviews. Formulating corresponding norms and price standards for DTC telemedicine and improving the performance incentive evaluation mechanism is essential. Second, private DTC telemedicine should more strictly limit the first response time of health service providers. Finally, organizations should add advisory templates with clinical guidelines, such as significant symptoms and dosage, on online platforms in public and private DTC telemedicine. In addition, they should reestablish scientific and reasonable doctor scheduling and collect information on the time distribution when patients initiate consultations.

## Appendix

Multimedia Appendix 1 Selection conditions and characteristics of SP cases. SP: standardized patient.

Multimedia Appendix 2 Level and ranking of provinces.

Multimedia Appendix 3 Condensed scripts and checklists.

Multimedia Appendix 4 Scoring method of patient-centeredness (PCC).

Multimedia Appendix 5 Quality of DTC telemedicine provided by public and private sectors. DTC: direct to consumer.

Multimedia Appendix 6 Effect of models on the quality of DTC telemedicine for different case. DTC: direct to consumer.

## Abbreviations

DTC: direct to consumer
IOM: Institute of Medicine
OLS: ordinary least squares
OR: odds ratio
PCC: patient-centeredness
PPPC: Patient Perception of Patient-Centeredness
SP: standardized patient
