# Supplementary material for: Comparing the Quality of Direct-to-Consumer Telemedicine Dominated and Delivered by Public and Private Sector Platforms in China: Standardized Patient Study
Source: J Med Internet Res. 2024 Nov 14;26:e55400. doi: 10.2196/55400 (PMC11605261; doi:10.2196/55400)
Supplement: Multimedia Appendix 1 [file jmir_v26i1e55400_app1.docx]

**Multimedia** **Appendix 1**

Table S1. Selection conditions and characteristics of SP cases

| Conditions | Characteristics | |
| --- | --- | --- |
|  | Urticaria | Childhood diarrhea |
| **Technical feasibility** |  |  |
| Can a trained SP portray the case? | Yes. The wheals will go away naturally, without raising a doctor’s suspicions. Besides, the symptoms of urticaria are simple and do not cause strong physical discomfort, so SP is easy to portray. | Yes. SP only needs to describe symptoms of a 2-year-old child consistent with a viral infection (including watery diarrhea without blood or mucus, no fever and change in behavior). |
| Do national or international guidelines exist for correct management or treatment? | Yes. Guideline for diagnosis and treatment of urticaria in China (2018) | Yes. Guideline for diagnosis and management of acute infectious diarrhea in children (Version 2020) |
| Can expected management be performed within one visit? | Yes. No follow-up visit is required. | Yes. No follow-up visit is required. |
| **Ethical acceptability** |  |  |
| Does the case choice minimize potential harm to fieldworkers? | No potential harm to fieldworkers. | No potential harm to fieldworkers. |
| Does the case require the involvement of children? | No. | No. SPs presented the case of a niece/nephew who was not present. |

Table S1. Selection conditions and characteristics of SP cases (Continued)

| Conditions | Characteristics | |
| --- | --- | --- |
|  | Urticaria | Childhood diarrhea |
| **Appropriateness to context and research question** |  |  |
| Is the case appropriate to the study objective? | Yes. Dermatosis is one of the main diseases in Internet diagnosis and treatment in China. | Yes. Diarrhoea remains one of the leading causes of child morbidity and mortality worldwide. |
| Do stakeholders agree the case is a ‘fair test’? | They all agree. | They all agree. |
| Is the case applicable to all health facilities and regions in the study? | Yes, the research team has screened hospitals and doctors that meet the requirements of the study. | Yes, the research team has screened hospitals and doctors that meet the requirements of the study. |
| Does the case represent a public health concern? | In the past 30 years, the incidence of allergic diseases has increased at least threefold and has reached as high as 20%. The incidence of urticaria in China has also continued to rise, with 9% to 20% of people ever suffering from urticaria. | As a global public health problem, diarrhea is the second leading cause of death in children under 5 years of age and nearly 2 million children die from diarrhoea every year. |
| Does the case match local epidemiology? | Dermatosis is one of the earliest telemedicine diseases because of its visual diagnosis. Dermatology consultation volume ranked top 5 in Beijing tertiary hospitals, and dermatology consultation volume increased significantly after the epidemic. | The pediatrics was one of the most common department in China. |
