# Supplementary material for: Comparing the Quality of Direct-to-Consumer Telemedicine Dominated and Delivered by Public and Private Sector Platforms in China: Standardized Patient Study
Source: J Med Internet Res. 2024 Nov 14;26:e55400. doi: 10.2196/55400 (PMC11605261; doi:10.2196/55400)
Supplement: Multimedia Appendix 2 [file jmir_v26i1e55400_app2.docx]

**Multimedia Appendix 2**

Table S2. The level and ranking of provinces.

| **Level** | **Province** | **Economic development level** | | **Health development status** | | **Comprehensive ranking** |
| --- | --- | --- | --- | --- | --- | --- |
|  |  | **Real GDP** | **Ranking** | **Life expectancy** | **Ranking** |  |
| Level Ⅰ | Beijing | 164220 | 1 | 80.18 | 2 | 1.5 |
|  | Shanghai | 157279 | 2 | 80.26 | 1 | 1.5 |
|  | Jiangsu | 123607 | 3 | 76.63 | 5 | 4 |
|  | Zhejiang | 107624 | 4 | 77.73 | 4 | 4 |
|  | Tianjin | 90371 | 7 | 78.89 | 3 | 5 |
|  | Guangdong | 94172 | 6 | 76.49 | 6 | 6 |
|  | Fujian | 107139 | 5 | 75.76 | 12 | 8.5 |
|  | Shandong | 70653 | 10 | 76.46 | 7 | 8.5 |
|  | Chongqing | 75828 | 9 | 75.7 | 13 | 11 |
|  | Liaoning | 57191 | 15 | 76.38 | 8 | 11.5 |
| Level Ⅱ | Hainan | 56507 | 16 | 76.3 | 9 | 12.5 |
|  | Hubei | 77387 | 8 | 74.87 | 18 | 13 |
|  | Anhui | 58496 | 13 | 75.08 | 15 | 14 |
|  | Shaanxi | 66649 | 12 | 74.68 | 21 | 16.5 |
|  | Hunan | 57540 | 14 | 74.7 | 20 | 17 |
|  | Inner Mongolia | 67852 | 11 | 74.44 | 23 | 17 |
|  | Sichuan | 55774 | 18 | 74.75 | 19 | 18.5 |
|  | Jilin | 43475 | 28 | 76.18 | 10 | 19 |
|  | Henan | 56388 | 17 | 74.57 | 22 | 19.5 |
|  | Heilongjiang | 36183 | 30 | 75.98 | 11 | 20.5 |
|  | Hebei | 46348 | 26 | 74.97 | 16 | 21 |
| Level Ⅲ | Guangxi | 42964 | 29 | 75.11 | 14 | 21.5 |
|  | Shanxi | 45724 | 27 | 74.92 | 17 | 22 |
|  | JIangxi | 53164 | 21 | 74.33 | 24 | 22.5 |
|  | Ningxia | 54217 | 20 | 73.38 | 25 | 22.5 |
|  | Xinjiang | 54280 | 19 | 72.35 | 26 | 22.5 |
|  | Qinghai | 48981 | 22 | 69.96 | 29 | 25.5 |
|  | Guizhou | 46433 | 25 | 71.1 | 28 | 26.5 |
|  | Xizang | 48902 | 23 | 68.17 | 31 | 27 |
|  | Yunnan | 47944 | 24 | 69.54 | 30 | 27 |
|  | Gansu | 32995 | 31 | 72.23 | 27 | 29 |
