# Supplementary material for: Comparing the Quality of Direct-to-Consumer Telemedicine Dominated and Delivered by Public and Private Sector Platforms in China: Standardized Patient Study
Source: J Med Internet Res. 2024 Nov 14;26:e55400. doi: 10.2196/55400 (PMC11605261; doi:10.2196/55400)
Supplement: Multimedia Appendix 3 [file jmir_v26i1e55400_app3.docx]

**Multimedia Appendix 3**

Table S3. Condensed scripts and checklists

| Category | Urticaria | | Childhood diarrhea |
| --- | --- | --- | --- |
| **Opening Statement** | Hello doctor, I’ve been getting red rashes all over my body. Could you tell me what’s wrong with them? | Hello, doctor. My little nephew/daughter started having loose stools yesterday. I’m not sure what's going on, so I thought I'd come to get some advice on how to handle it. | |
| **Onset and duration of disease** | The illness began two weeks ago, which once in two or three days in the first week, and worsened in the past week, four or five times a week. It generally happened in the morning or after breakfast, and faded after a few hours. There was no symptoms at present. | The child have had loose bowels 1 day ago and had four or five bowel movements a day. | |
| **Cardinal symptom** | 1. The rash was a little hard and raised after grasping, its shape was uncertain, flat, drums, and not pointed.  2. Mainly distributed on legs and arms  3. No local edema and pain  4. Itchy  5. Irregular recurrence with no obvious pigmentation. | Stool was yellow dilute water, no mucus, pus and blood, and no special odor. | |
| **Concomitant symptom** | No fever, nausea, vomiting, abdominal pain, diarrhea, sore throat, chest tightness, suffocation, dyspnea, etc | 1. Vomited 2/3 times  2. No fever and no bloating, etc.  3. No significant changes in food intake and spirit.  4. Cry more, pee less | |
| **Predisposing factor** | No. | No. | |
| **Previous medical experience** | No medical attention and medication | No medical attention and medication | |
| **Past medical history** | No. | No. | |
| **Family history** | No. | No. | |
| **Peroration** | What the hell was wrong with me?  Do I need any medicine or ointment?  Was there anything I should be aware of? | What kind of illness did the child have?  Do I need to give the baby some medicine?  Was there anything should pay attention to the child ? | |

The following are examples of specific consultations in English and Chinese:

**Urticaria Case 1**

7/20/2022 11:56 SP: Hello doctor, I’ve been getting red rashes all over my body. Could you tell me what’s wrong with them?

7/20/2022 11:56 SP：医生您好，我最近这段时间身上长红色大疙瘩，很痒，想咨询您看看是怎么回事。

7/20/2022 17:25 D: Any photos of the rash?

7/20/2022 17:25 D：您好，有皮疹的照片吗？

7/20/2022 17:26 SP: Photo.

7/20/2022 17:26 SP：提供图片。

7/20/2022 23:47 D: How long will this rash last? When did the first rash appear? Have you been taking any other medications for other illnesses recently?

7/20/2022 23:47 D：这个皮疹持续会超过24小时吗？第一次起皮疹离现在有多久了？最近有没有因为其他病在吃药？

7/20/2022 23:49 SP: The rash clears up within a few hours. It’s been almost two weeks since the first one. I haven’t taken any medication recently.

7/20/2022 23:49 SP：一般起了之后几个小时就消了。第一次起到现在差不多两周了。最近没吃什么药。

7/21/2022 00:54 D: (1) Do you have any underlying medical conditions? Are you pregnant or breastfeeding? (2) You have hives, and it’s important to see a doctor if you’re experiencing chest tightness, shortness of breath, or other discomforts.

7/21/2022 00:54 D：（1）有没有什么基础病？有没有怀孕哺乳？（2）你这个是荨麻疹，如果有胸闷呼吸困难等不适要及时就诊。

7/21/2022 06:26 SP: There’s no underlying medical condition, and no chest tightness or shortness of breath.

7/21/2022 06:26 SP：没有基础病，目前没有胸闷呼吸困难。

7/21/2022 12:40 D: If the rash comes back, you can take Ebastine Tablets, one tablet once or twice a day.

7/21/2022 12:40 D：如果皮疹反复，可以吃开思亭，每天一到两次，每次一片。

7/21/2022 13:06 SP: Where can I buy this medicine?

7/21/2022 13:06 SP：好的医生，这个药在哪里能买呢？

7/21/2022 13:33 D: You can get it at the pharmacy, but if not, you can also buy Loratadine, which should only be taken as one tablet a day.

7/21/2022 13:33 D：药店就有，如果没有，也可以买氯雷他定，氯雷他定只能每天吃一片。

7/21/2022 13:34 SP: Is there anything else I should be aware of?

7/21/2022 13:34 SP：好的医生，那我平时还有什么需要注意的吗？

7/21/2022 15:33 D: It’s best to avoid high-protein foods like seafood, fish, shrimp, crab, beef, lamb, eggs, milk, pineapple, mango, and durian. Once the rash has gone, it’s best to eat only one of these in a day. If you do get a rash, eat as little as possible in the future.

7/21/2022 15:33 D：这几天高蛋白食物如海鲜鱼虾蟹牛羊肉鸡蛋牛奶菠萝芒果榴莲最好先不吃，以后没有皮疹了，如果想吃，一天内最好只吃一种，如果吃了有发疹，以后就尽量少吃。

7/21/2022 15:33 SP: Okay, I get it. Thanks, doctor.

7/21/2022 15:33 SP：好的好的我明白了，谢谢医生。

7/21/2022 15:33 D: If the rash is large or if you’re experiencing discomfort like chest tightness or difficulty breathing, it’s important to seek medical attention.

7/21/2022 15:33 D：如果皮疹面积很大，或者有胸闷、呼吸困难等不适要及时就诊。

END

**Urticaria Case 2**

7/22/2022 11:31 SP: Hello doctor, I’ve been getting red rashes all over my body. Could you tell me what’s wrong with them?

7/20/2022 11:56 SP：医生您好，我最近这段时间身上长红色大疙瘩，很痒，想咨询您看看是怎么回事。

7/22/2022 12:56 D: Hello! Could you tell me how long the rash has been there? How long does it take for the red rash to go away once it appears? Could you possibly post a couple of pictures of the rash?

7/22/2022 12:56 D：你好！皮疹多少时间了？红色皮疹出现以后多少时间会消退？能发几张皮疹的图片看一下吗？

7/22/2022 15:40 SP: (1) Photo. (2) It’s been almost two weeks now. The rash usually clears up within a couple of hours of starting.

7/22/2022 15:40 SP：（1）提供照片。（2）差不多两周了。一般起了之后几个

小时就消了。

7/22/2022 17:11 D: This is hives. You can take an oral anti-allergy medication, like Loratadine, once a day.

7/22/2022 17:11 D：这个是荨麻疹，可口服抗过敏药，开瑞坦一天一片。

7/22/2022 17:18 SP: Where I can buy this medicine?

7/22/2022 17:18 SP：好的医生，这个药哪里能买呢。

7/22/2022 17:20 D: You’ll find them in hospitals and pharmacies.

7/22/2022 17:20 D：医院、药店都有。

7/22/2022 20:26 SP: Is there anything I should be aware of ?

7/22/2022 20:26 SP：请问我平时有什么要注意的吗？

The doctor hadn’t answered until the end of the visit.

直到问诊结束医生未回复。

**Urticaria Case 3**

7/6/2022 10:03 SP: Hello doctor, I’ve been getting red rashes all over my body. Could you tell me what’s wrong with them?

7/6/2022 10:03 SP：医生您好，我最近这段时间身上长红色大疙瘩，很痒，想咨询您看看是怎么回事。

7/7/2022 12:50 D: It could be a keloid. You can send me a picture. If it is, you’ll need to apply ointment and have a X-ray or laser treatment to get better.

7/7/2022 12:50 D：大疙瘩的话有可能是瘢痕疙瘩，你要发图片给我们看。如果是的话，要涂药膏，打浅深X光或者点阵激光才能够好转。

7/7/2022 14:23 SP: Photo.

7/7/2022 14:23 SP：提供照片。

7/7/2022 23:21 D: (1) It might be hives. You can take Compound Glycyrrhizin Tablets, two tablets three times a day. You could also try Loratadine, which you can take once a day to see if that helps. If that doesn’t do the trick, another Levocetirizine Hydrochloride Oral Solution at night might be better. (2) If you think you might have a co-infection, have a blood test done to find out. If you do, take some oral anti-infective medication.

7/7/2022 23:21 D：（1）可能是荨麻疹。你可以吃美能片，每天三次，每次两片。或者吃枸地氯雷他定片，每天吃一片，看看能不能缓解。如果不能缓解，晚上再喝一支左西替利嗪口服液可能会好一些。（2）抽血看看有没有合并感染，如果有感染的话，要口服一些抗感染的药物。

7/7/2022 23:23 SP: Where I can buy these medicine?

7/7/2022 23:23 SP：这些药哪里能买呢？

The doctor hadn’t answered until the end of the visit.

直到问诊结束医生未回复。

**Childhood Diarrhea Case 1**

7/3/2022 19:07 SP: Hello, doctor. My little daughter started having loose stools yesterday. I’m not sure what’s going on, so I thought I'd come to get some advice on how to handle it.

7/3/2022 19:07 SP：医生您好，我家小侄女，从昨天开始有点拉肚子，不知道咋回事儿，所以来咨询您看看该怎么处理。

7/3/2022 19:20 D: Hello, could you tell me what kind of stools children have? Is it loose or watery? Any fever or vomiting?

7/3/2022 19:20 D：您好，小孩大便是什么样的？稀烂便还是水样？有没有发热或呕吐？

7/3/2022 19:21 SP: All the poop is thin water. The child has no fever. Threw up two or three times since yesterday.

7/3/2022 19:21 SP：拉的都是稀水。没有发热，从昨天到现在吐了两三次。

7/3/2022 19:25 D: Has the child had anything unusual to eat recently? Any medication the child is taking? Is it vomiting with or without medication? Did he/she have any stomach discomfort?

7/3/2022 19:25 D：最近有吃特殊的东西吗？吃药没？呕吐是吃药的时候呕吐还是没吃药的时候？什么情况下呕吐？有没有肚子疼？

7/3/2022 21:23 SP: She didn’t eat anything special or take any medication. I didn’t see the child throw up. She shouldn’t have a stomachache. It seems like the child is crying a little more, and I’m not sure if it’s a tummy ache.

7/3/2022 21:23 SP：没有吃特殊的东西，也没吃药。没注意到什么情况下吐。应该没有肚子疼。感觉哭闹多了点，不知道是不是肚子疼。

7/3/2022 21:34 D: It might be worth considering whether the discomfort is causing it. How often does she have watery poop? Have she had it checked out by a doctor? How is she feeling mentally?

7/3/2022 21:34 D：有可能不舒服引起。每天都拉几次水样便？有没有去医院看？精神反应怎么样？

7/3/2022 22:13 SP: The child has had four or five bowel movements since yesterday, and her mental state seems to be about the same as before, just crying more. I didn’t take her to the hospital.

7/3/2022 22:13 SP：从昨天到现在拉了4-5次，精神状态感觉和之前差不多，就是哭闹多些。没有去医院看。

7/3/2022 22:20 D: (1) Crying is often linked to dehydration due to diarrhea and vomiting, and electrolyte imbalances can also occur. (2) You can also give your child Montmorillonite powder and Saccharomyces boulardii Sachets, as well as oral rehydration salts. (3) Your child hasn’t been eating well for the past two days, so it’d be best to go to the hospital and have your child evaluated by a doctor in person to see if she should be given medication or IV fluids. If your child is vomiting and repeated diarrhea for too long, it can make the problem worse. It’s best to go to the hospital as soon as possible to get it under control.

7/3/2022 22:20 D：（1）哭闹多跟又拉又吐导致脱水有关，有可能电解质紊乱。（2）可以给小孩吃蒙托石散和布拉氏酵母菌散以及口服补液盐。（3）估计你小孩这两天吃得也不好，建议你最好去医院看一下，让医生当面评估后看看是吃药还是打点滴。长时间反复拉水和呕吐对孩子不好，容易导致病情加重，趁现在赶紧去医院看下治疗，及早控制好。

7/3/2022 22:53 SP: What could the child be suffering from? Where can I get these medications? I have cefixime at home. My neighbor’s child had diarrhea before. They said the child recovered quickly after eating. Can my child take this?

7/3/2022 22:53 SP：那孩子可能是什么病呢？这些药哪里可以买呢？我家里有头孢克肟，邻居家孩子之前拉肚子吃了很快就好了，我们孩子可以吃这个吗？

7/3/2022 23:32 D: Your child doesn’t need to take a cefixime in this case. She need to take Montmorillonite powder for it to work.

7/3/2022 23:32 D：你这个不一定要吃头孢，蒙托石散必须要吃才行。

7/3/2/22 23:35 SP: We’ll be seeing the doctor as soon as we can. Is there anything we should be aware of regarding that child?

7/3/2/22 23:35 SP：我们尽快去看病，那小孩子有什么需要注意的吗？

7/3/2022 23:37 D: (1) Your child may have gastroenteritis, and diet plays a big part in it. It’s probably caused by eating unclean things. This can be pretty serious or pretty mild. If your child is acting out or irritable, it’s probably because they’re dehydrated. In that case, it’s probably best to go to the hospital right away. (2) If you can, give your child plenty of water with some salt in it. It’s probably best to take it to the hospital, where the doctor can evaluate it and prescribe medication to treat it.

7/3/2022 23:37 D：（1）你小孩可能是肠胃炎，跟饮食关系很大，很可能是吃了不干净的东西造成。这个有重有轻，如果出现精神不好或者烦躁不安，说明脱水严重，还是及时要去医院看。（2）能喝水的话给孩子多喝点，里面放点盐。但是感觉还是带去医院安全一些，医生会给你评估开药治疗。

7/4/2022 00:11 SP: Tanks, doctor.

7/4/2022 00:11 SP：嗯嗯，谢谢医生。

END

**Childhood Diarrhea Case 2**

7/23/2022 11:16 SP: Hello, doctor. My little nephew started having loose stools yesterday. I’m not sure what’s going on, so I thought I'd come to get some advice on how to handle it.

7/23/2022 11:16 SP：医生您好，我家小侄子，从昨天开始有点拉肚子，不知道咋回事儿，所以来咨询您看看该怎么处理。

7/23/2022 11:50 D: How often does your child have a bowel movement during the day? What shape are we talking about here? Any fever or vomiting? Any abdominal discomfort? How is the child’s mood?

7/23/2022 11:50 D：每天几次？什么形状？发热或呕吐吗？有没有腹痛？精神怎么样？

7/23/2022 12:34 SP: Since this time yesterday the child has pooped about four or five times, and the poop has been thin and watery. There is no fever, and he has vomited two or three times. Maybe a bit of abdominal pain, feel a bit more crying today, not good to bring. His spirit is more or less the same as before.

7/23/2022 12:34 SP：从昨天这会儿到现在拉了大概四、五次，拉的都是稀水。没有发热，吐了两三次。可能有点腹痛，感觉今天哭闹有点多，不太好带。精神和之前差不多。

7/23/2022 12:45 D: Get some Montmorillonite powder and probiotics to take. If your child keeps vomiting and can’t nurse or eat, it’s a good idea to see a doctor. It’s important to see the child in person to assess his mental status and look out for signs of dehydration. If he eats milk and food without vomiting and can drink water without vomiting, you can start him on montelukast. Give him half a packet three times a day on an empty stomach. Take it for two or three days to see if the diarrhea clears up. If it doesn't, he’ll need to check a stool routine and occult blood.

7/23/2022 12:45 D：买点蒙脱石散、益生菌吃。如果老是呕吐，不能吃奶或者吃饭，还是找医生看下。需要亲自看孩子，评估精神状态，警惕脱水。如果之后吃奶吃饭，没有呕吐，也能喝水不呕吐，就先买蒙脱石散，每次半包，一天三次，空腹吃。先吃两三天，看看腹泻能不能好，如果不能好，就需要查个大便常规+隐血。

7/23/2022 12:52 SP: Okay. I have cefixime at home. My neighbor’s child had diarrhea before. They said the child recovered quickly after eating. Can my child take this?

7/23/2022 12:52 SP：好的好的，医生还想问您，我邻居家的小孩，之前拉肚子的时候，他吃的是头孢克肟，很快就好了，不知道我家孩子能吃这个吗？

The doctor hadn’t answered until the end of the visit.

直到问诊结束医生未回复。

**Childhood Diarrhea Case 3**

8/1/2022 19:34 SP: Hello, doctor. My little nephew started having loose stools yesterday. I’m not sure what’s going on, so I thought I'd come to get some advice on how to handle it.

8/1/2022 19:34 SP：医生您好，我家小侄子，从昨天开始有点拉肚子，不知道咋回事儿，所以来咨询您看看该怎么处理。

8/1/2022 22:18 D: Just send the stool sample to the hospital to run some tests. Also, you can give your child Montmorillonite powder three times a day. Just take one half a packet on an empty stomach and the other half in 25ml of warm water.

8/1/2022 22:18 D：把大便送到医院去查一下大便常规。另外可以给孩子吃蒙脱石散和益生菌蒙脱石散，一天三次一次半包空腹吃，半包放25ml温开水。

8/1/2022 22:20 SP: What kind of disease does the child have? Where can I get these medications?

8/1/2022 22:20 SP：您看孩子这大概是什么病呀？这些药要去哪里买呢？

8/2/2022 07:33 D: You can get these medicines at your local pharmacy.

8/2/2022 07:33 D：这些药在药店里都可以买得到的。

8/2/2022 08:32 SP: I also have cefixime at home. My neighbor’s child had diarrhea before. They said the child recovered quickly after eating. Can my child take this?

8/2/2022 08:32 SP：好嘞，我家里还有头孢克肟，是之前邻居家孩子拉肚子时候吃的，说是吃了很快就好了，您看我家孩子能吃这个药不？

8/2/2022 13:06 D: Not for now.

8/2/2022 13:06 D：暂时不需要。

8/2/2022 13:13 SP: Okay, I see. Is there anything the kid should be aware of？

8/2/2022 13:13 SP：好的明白了，那孩子平时有什么需要注意的吗？

The doctor hadn’t answered until the end of the visit.

直到问诊结束医生未回复。
