# Supplementary material for: Comparing the Quality of Direct-to-Consumer Telemedicine Dominated and Delivered by Public and Private Sector Platforms in China: Standardized Patient Study
Source: J Med Internet Res. 2024 Nov 14;26:e55400. doi: 10.2196/55400 (PMC11605261; doi:10.2196/55400)
Supplement: Multimedia Appendix 4 [file jmir_v26i1e55400_app4.docx]

**Multimedia Appendix 4**

Table S4. The scoring method of PCC

| Dimension | Description | Scoring |
| --- | --- | --- |
| **PCC1** | The doctor made you feel comfortable expressing your symptoms and fears. | Using 5-point Likert scale (e.g. strongly agree, agree, uncertain, disagree, strongly disagree); ranging from 5 to 1. |
| **PCC2** | Home address | 1 if the physician obtained the information, 0 otherwise. Calculate the total score of all the essential items. |
|  | Family history |  |
|  | employment status |  |
| **PCC3** | You agreed with the doctor about the disease. | Using 5-point Likert scale (e.g. strongly agree, agree, uncertain, disagree, strongly disagree); ranging from 5 to 1. |
|  | The doctor fully explained to you the problem of your illness. | Using 5-point Likert scale (e.g. strongly agree, agree, uncertain, disagree, strongly disagree); ranging from 5 to 1. |
|  | The doctor fully explained the treatment plan to you. | Using 5-point Likert scale (e.g. strongly agree, agree, uncertain, disagree, strongly disagree); ranging from 5 to 1. |
|  | Did the doctor discuss your role when considering disease management? | 1 if the doctor discussed patient’s role, 0 otherwise. |
|  | Did you like this doctor? | 1 if SP liked the doctor, 0 otherwise. |
