# Supplementary material for: Comparing the Quality of Direct-to-Consumer Telemedicine Dominated and Delivered by Public and Private Sector Platforms in China: Standardized Patient Study
Source: J Med Internet Res. 2024 Nov 14;26:e55400. doi: 10.2196/55400 (PMC11605261; doi:10.2196/55400)
Supplement: Multimedia Appendix 5 [file jmir_v26i1e55400_app5.docx]

**Multimedia Appendix 5**

Table S5. Quality of DTC telemedicine provided by public and private sector

| Variables | Total (n=321) median(IQR) / n(%) | Public Sector (n=175) median(IQR) / n(%) | Private Sector (n=146) median(IQR) / n(%) | *Z* | *P* Value |
| --- | --- | --- | --- | --- | --- |
| **Effectiveness and safety** |  |  |  |  |  |
| Adherence to checklist | 25.00(16.67~33.33) | 16.67(8.33~27.78) | 29.17(22.22~38.89) | -8.120 | ＜.001 |
| Accurate diagnosis | 266(82.9) | 132(75.4) | 134(91.8) | -3.866 | ＜.001 |
| Appropriate prescription | 70 (21.8) | 21(12.0) | 49(33.6) | -4.651 | ＜.001 |
| Providing lifestyle modification advice | 263(81.9) | 128(73.1) | 135(92.5) | -4.474 | ＜.001 |
| **Patient-centredness** |  |  |  |  |  |
| PCC | 17.00(13.00~19.00) | 15.00(11.00~18.00) | 18.00(16.00~20.00) | -6.366 | ＜.001 |
| PCC1 | 4.00(3.00~5.00) | 4.00(3.00~4.00) | 4.00(4.00~5.00) | -4.716 | ＜.001 |
| PCC2 | 0.00(0.00~1.00) | 0.00(0.00~1.00) | 1.00(0.00~1.00) | -5.178 | ＜.001 |
| PCC3 | 12.00(10.00~14.00) | 11.00(8.00~13.00) | 13.00(12.00~14.00) | -5.821 | ＜.001 |
| **Timeliness** |  |  |  |  |  |
| Time waiting for the first response | 80.00(13.50~393.50) | 68.00(9.00~222.00) | 118.00(22.25~672.75) | -2.927 | .003 |
| Time waiting for each response | 118.73(21.33~328.25) | 106.23(28.28~283.90) | 126.17(17.75~398.05) | -0.410 | .68 |
| Time for consultation | 736.50(165.50~1407.25) | 679.50(197.50~1244.25) | 790.00(124.25~2334.50) | -1.592 | .11 |
| Total number of the doctor’s responses | 4.00(3.00~6.00) | 4.00(3.00~5.00) | 5.00(4.00~6.00) | -5.958 | ＜.001 |
| Total words in all of the doctor’s responses | 135.00(82.00~212.50) | 109.00(66.00~198.00) | 160.00(114.00~232.25) | -4.677 | ＜.001 |
| **Efficiency** |  |  |  |  |  |
| Total cost | 27.00(10.00~52.00) | 10.00(0.00~30.00) | 42.00(27.00~72.00) | -11.160 | ＜.001 |
