# Supplementary material for: Comparing the Quality of Direct-to-Consumer Telemedicine Dominated and Delivered by Public and Private Sector Platforms in China: Standardized Patient Study
Source: J Med Internet Res. 2024 Nov 14;26:e55400. doi: 10.2196/55400 (PMC11605261; doi:10.2196/55400)
Supplement: Multimedia Appendix 6 [file jmir_v26i1e55400_app6.docx]

**Multimedia Appendix 6**

Table S6. The effect of Models on the quality of DTC^a^ telemedicine for Urticaria.^b^

| Characteristics | Public sector adjusted β/OR^c^ (SE) | Private sector adjusted β/OR (SE) | *P* value |
| --- | --- | --- | --- |
| **Effectiveness and safety** |  |  |  |
| Adherence to the checklist | Reference | 14.38 (2.04) | .002 |
| Accurate diagnosis | Reference | 11.86 (11.89) | .02 |
| Appropriate prescription | Reference | 3.43 (1.43) | .003 |
| Providing lifestyle modification advice | Reference | 9.88 (5.78) | ＜.001 |
| **PCC^d^** |  |  |  |
| PCC | Reference | 3.09 (0.45) | .002 |
| PCC1 | Reference | 0.56 (0.10) | .004 |
| PCC2 | Reference | 0.23 (0.14) | .18 |
| PCC3 | Reference | 2.29 (0.42) | .005 |
| **Timeliness** |  |  |  |
| Time waiting for the first response | Reference | 541.22 (69.41) | .001 |
| Time waiting for each response | Reference | 233.59 (107.32) | .10 |
| Time for consultation | Reference | 849.75 (138.31) | .004 |
| Total number of the doctor’s responses | Reference | 0.83 (0.28) | .04 |
| Total words in all of the doctor’s responses | Reference | 54.12 (24.71) | .10 |
| **Efficiency** |  |  |  |
| Total cost | Reference | 46.20 (3.85) | ＜.001 |

^a^DTC: direct to consumer.

^b^The variables were controlled as follows: region, type of institution, physician gender, physician title, and timing of the visit, and SE was the clustering robust standard error.

^c^OR: odds ratio.

^d^PCC: patient-centeredness.

Table S7. The effect of Models on the quality of DTC^a^ telemedicine for Childhood diarrhea.^b^

| Characteristics | Public sector adjusted β/OR^c^ (SE) | Private sector adjusted β/OR (SE) | *P* value |
| --- | --- | --- | --- |
| **Effectiveness and safety** |  |  |  |
| Adherence to the checklist | Reference | 15.40 (2.78) | .005 |
| Accurate diagnosis | Reference | 3.68 (1.66) | .004 |
| Appropriate prescription | Reference | 3.20 (2.26) | .10 |
| Providing lifestyle modification advice | Reference | 5.51 (2.12) | ＜.001 |
| **PCC^d^** |  |  |  |
| PCC | Reference | 3.54 (0.50) | .002 |
| PCC1 | Reference | 0.62 (0.14) | .01 |
| PCC2 | Reference | 0.47 (0.26) | .14 |
| PCC3 | Reference | 2.45 (0.57) | .01 |
| **Timeliness** |  |  |  |
| Time waiting for the first response | Reference | 396.14 (157.12) | .07 |
| Time waiting for each response | Reference | 30.61 (35.16) | .44 |
| Time for consultation | Reference | 660.93 (268.5) | .07 |
| Total number of the doctor’s responses | Reference | 1.75 (0.35) | .008 |
| Total words in all of the doctor’s responses | Reference | 56.04 (10.32) | .006 |
| **Efficiency** |  |  |  |
| Total cost | Reference | 32.96 (5.29) | .003 |

^a^DTC: direct to consumer.

^b^The variables were controlled as follows: region, type of institution, physician gender, physician title, and timing of the visit, and SE was the clustering robust standard error.

^c^OR: odds ratio.

^d^PCC: patient-centeredness.
